# Atypical presentations and rare metastatic sites of renal cell carcinoma: a review of case reports

**DOI:** 10.1186/1752-1947-5-429

**Published:** 2011-09-02

**Authors:** Petros Sountoulides, Linda Metaxa, Luca Cindolo

**Affiliations:** 1 Department of Urology, General Hospital of Veria, Asomata Verias 59100, Veria, Greece; 2 Department of Radiology, General Hospital of Veria, Asomata Verias 59100, Veria, Greece; 3 Department of Urology, S Pio da Pietrelcina Hospital, Via C De Lellis 1, I-66054, Vasto, Italy

## Abstract

Renal cell carcinoma is a potentially lethal cancer with aggressive behavior and a propensity for metastatic spread. Due to the fact that the patterns of metastases from renal cell carcinomas are not clearly defined, there have been several reports of cases of renal cell carcinoma associated with rare metastatic sites and atypical presenting symptoms. The present review focuses on these atypical rare clinical presentations of renal cell carcinomas both at the time of diagnosis of the primary tumor but also in the years after radical nephrectomy.

## Introduction

Renal cell carcinoma (RCC) is a lethal tumor that accounts for approximately 3% of all adult malignancies and is associated with approximately 13,000 deaths annually [1]. The introduction and widespread use of sophisticated imaging modalities has resulted in a significant increase in the incidental detection of kidney tumors. Nowadays more than 70% of all renal cancer cases are "screen detected" as incidental findings on imaging studies obtained for unrelated reasons [2]. Therefore the classical teaching that renal cancer presents with signs and symptoms such as hematuria, flank pain and palpable mass is more of the exception rather than the rule. This trend has also resulted in a significant shift in the staging of renal cancer since lesser cases initially present with advanced metastatic disease and more cases of renal tumors are confined to the kidney at the time of diagnosis.

Still, renal cancers have a strong tendency to metastasize following occasionally unpredictable patterns of spread. There have been several reports of late metastases from RCC even decades after potentially curative surgical excision of the primary tumor. There is evidence that distant metastatic disease will eventually develop in about one out of three patients with RCC and in these cases the disease is considered incurable. Even despite recent therapeutic advances in the management of metastatic renal cancer such as immunotherapy and mTOR kinase inhibitors, long-term survival in patients with metastatic RCC is limited to months [3-5].

With regard to the histologic subtypes of RCC and their relationship to prognosis, the most common subtype, which is clear cell renal cancer, accounts for 70-80% of all RCCs. Chromophobe cell carcinoma accounts for only 3-5% of all RCCs and carries a better prognosis than clear cell RCC with a five-year survival rate between 92-94% [6,7]. The pathologic stage of RCC at the time of presentation has been demonstrated to correlate most closely with survival [8].

### Metastatic pathway in RCC

The development of metastatic disease is a sequential process where cancer cells depart from the primary tumor via the blood supply or lymphatic chain and deposit at proximal or distant sites. This metastatic pathway is not always predictable and certainly not for renal cancer, which is notorious for its complex lymphatic drainage. However there is a predilection for certain sites, meaning that these sites are usually the first occupied by cancer cells [9]. Moreover, there has been evidence in support of an early dissemination model, where metastasis occurs early in the lifecycle of cancer cells.

In an experimental study, engineered untransformed mouse mammary cells were found to express inducible oncogenes transgenes that were able to bypass the primary site and show up at secondary metastatic sites [10]. In another animal study, Kaplan *et al*. also showed that cancer cells in mice models might have instructed bone marrow cells to migrate to pre-selected organs in order to establish a hospitable environment. This event preceded the appearance of cancer cells by four to six days and micrometastatic colonies formed five days later [11]. These studies might explain the unpredictable metastatic pattern of renal tumors and account for the late appearance of metastatic disease in organs and sites that are considered outside of the "usual" metastatic pathway of RCC.

## Rare metastatic sites of renal cell cancer

A Medline/PubMed search for articles in English (mostly case series and case reports) on rare metastatic sites of renal carcinomas was performed. In our search we considered as rare all sites that were anatomically distal to the kidney and outside the considered usual chain of metastatic spread of renal tumors. For that reason we excluded all sites of common metastases from renal tumors, including the lungs, adrenals, intestines and brain and most intra-abdominal organs, and only included rare metastatic sites outside the abdomen.

### Head and neck

RCC is the third most frequent neoplasm to metastasize to the head and neck region preceded only by breast and lung cancer. Despite being reported infrequently, head and neck region metastases may be linked to RCC in up to 15% of cases [12]. The nose and paranasal sinuses are most commonly affected, followed by the oral cavity.

### Orbit

Ocular metastases from RCCs are extremely rare. During the last five years only 19 cases have been reported. Ocular metastases are more likely to involve the iris, ciliary body and choroids, although eyelid, lacrimal sac and orbital metastases have also been described [13-16]. Among those 19 cases, 13 involved men and only three involved women. In three cases there was no mention of the gender. The mean age at initial diagnosis was 50 years. In seven cases the eye or orbital metastasis was the first manifestation of a previously unknown RCC, while in 10 cases there was a history of nephrectomy for RCC (one month to 17 years before the diagnosis of the ocular metastatic lesion). In two cases there was no mention of the previous medical history. The patients presented with several symptoms depending on the localization of the tumor, such as proptosis, diplopia, eye vision difficulties, cataract, upper lid tumor, and epiphora. The final diagnosis occurred after excision biopsy which revealed metastatic RCC. In Table 1 all cases with metastases to the orbit are presented in detail.

**Table 1:** Cases of RCC metastases to the orbit

Patient sex age at diagnosis	Location	Treatment of primary tumor	Interval between diagnosis of primary tumor and metastasis (months)	Diagnosis of metastasis before primary treatment	Presenting symptoms	Metastases to other sites
m/54	Uvea	Nephrectomy	36	no	Eye vision problem	-

NA	Bilateral lacrimal gland	NA	NA	NA	NA	-

m/43	Choroidal and conjunctival	Nephrectomy	12	no	Left orbital pain	-

NA	Choroidal, ocular, extraocular, vitreous	Nephrectomy	96	no	Cataract	-

m/52	Choroidal	Nephrectomy	24	no	Eye vision problem	Lung

m/63	Eyelid	Nephrectomy/chemotherapy	NA	no	Upper lid tumor	Lung

m/58	Inferior rectus muscle	-	Metastasis first	yes	Proptosis and diplopia	-

f/NA	Lacrimal sac	Nephrectomy	NA	NA	Epiphora	-

m/73	Orbit	Nephrectomy	204	no	Epistaxis	Nasal cavity, ethmoidal sinus

m/67	Iris	Nephrectomy	NA	no	NA	-

m/58	Orbit	-	Metastasis first	yes	NA	-

f/23	Orbit	Nephrectomy	one	no	NA	-

f/60	Orbit	-	Metastasis first	yes	Proptosis	-

m/69	Orbit	-	Metastasis first	yes	Proptosis	-

m/66	Eyelid	Nephrectomy	15	no	NA	Lung, skin, brain

m/47	Eyelid	Nephrectomy	33	no	NA	Abdomen, skin, lung

m/59	Retroocular	-	Metastasis first	yes	Proptosis and diplopia	-

m/72	Conjuctival	-	Metastasis first	yes	Ulcerated lesion	-

m/70	Orbit	-	Metastasis first	yes	Cataract	-

f: female; m: male; NA: not available

### Parotid gland

Major salivary gland metastases from distant primary tumors are very uncommon. Parotid metastatic lesions may originate from hepatocellular carcinoma, squamous cell carcinoma, melanoma, retinoblastoma, carcinoma of the breast, urachus, prostate, stomach, lungs or kidneys. An extensive literature search revealed a total of 26 cases of RCC metastatic to the parotid gland. In 14 of these patients, parotid metastasis was the initial sign of the kidney tumor. In the other 12 cases, parotid metastasis occurred after nephrectomy for RCC, at a time interval ranging from months to years [17-22].

To the best of our knowledge, the longest interval from nephrectomy to solitary parotid metastasis was 10 years. The most common presenting symptom was the presence of a palpable parotid mass, while in one case facial paralysis was the presenting symptom. In all cases fine-needle aspiration (FNA) biopsy was diagnostic. Some cases are presented in detail in Table 2[17-26].

**Table 2:** Cases of RCC metastases to the salivary glands

Patient sex and age at diagnosis	Location	Treatment of primary tumor	Interval between diagnosis of primary tumor and metastasis (months)	Diagnosis of metastasis before primary treatment	Presenting symptoms
NA	parotid gland	NA	NA	NA	facial nerve palsy

f/58	parotid gland	-	Metastasis first	yes	painless mass

f/76	parotid gland	Nephrectomy	108	no	painless mass

f/62	parotid gland	Nephrectomy	60	no	painless mass

NA	parotid gland	NA	NA	NA	painless mass

NA	parotid gland	NA	NA	NA	painless mass

NA	parotid gland	Nephrectomy	NA	no	painless mass

NA	parotid gland	Nephrectomy	120	no	painless mass

NA	parotid gland	Nephrectomy	24	NA	painless mass

m/66	parotid gland	Nephrectomy	18	NA	painless mass

m/69	parotid gland	Nephrectomy	24	no	painless mass

f/59	parotid gland	Nephrectomy	120	no	painless mass

f/61	parotid gland	-	Metastasis first	yes	painless mass

m/83	submaxillary gland	Nephrectomy	120	no	NA

f/52	minor salivary gland	-	Metastasis first	yes	NA

f/61	submandibular glands	Nephrectomy	84	no	Painless submandibular swelling

f: female; m: male; NA: not available

With regard to solitary submaxillary gland metastasis from RCC, we were able to retrieve three cases. These involved an 83-year-old man where metastatic tumor presented 10 years after primary treatment; a 52- year-old woman with a growing mass at the base of her tongue (minor salivary gland) and no known history of renal cancer and a 61-year-old patient who presented seven years after primary treatment, with metastasis to both the submandibular glands and the thyroid [26,27].

### Nasal and paranasal cavities

The nose is another very uncommon site for metastatic RCC. Approximately 50 cases of nasal recurrences of RCC have been reported in the literature. The maxillary sinuses are the paranasal sinuses most commonly afflicted by metastatic tumors to the sinonasal region, followed in frequency by the ethmoid, frontal, and sphenoid, while there is only one reported case of an isolated metastasis to the nasal septum.

Tumor involvement of the paranasal sinuses and nasal fossae appears to occur via the hematogenous route through the Batson's paravertebral venous plexus. This is an anastomotic network of avalvular veins surrounding the bone marrow and vertebras, connected with pelvic, intercostal, azygos and cava veins, therefore allowing tumor seeding in both a caudal direction toward the pelvis and a cranial direction to the calotte. Increased intra-abdominal or intrathoracic pressure causes an increased flow to the paravertebral plexus, from which venous sinuses in the calotte, and retrogradely the pterygoid plexus, are reached before arriving at the paranasal sinuses. This theory explains how tumor cells may escape the pulmonary capillary filter and how renal, pulmonary, genitourinary, or breast tumors can metastasize into the paranasal sinuses [28].

The most frequent patients' complaints were nasal obstruction, swelling and pain, although epistaxis is the most alarming symptom because of the high vascular stroma of these metastatic deposits. The high vasculature of RCC resulting in bleeding is probably caused by the fact that the von Hippel-Lindau gene mutation causes upregulation of hypoxia-induced factor 1α, which in turn leads to angiogenesis through vascular endothelial growth factor upregulation. In cases of uncontrollable bleeding, immediate surgical removal is mandatory [29].

In 15 of these cases there was no known history of renal mass, while the rest of the patients had previously undergone nephrectomy at a time interval ranging from eight to 18 years prior to the diagnosis of the metastatic lesion to the nasal or paranasal cavities. In the majority of the cases there were also synchronous metastases to other parts of the body like small intestine, lungs and thyroid glands. The definitive diagnosis (Table 3) was based on pathology of the lesion, supplemented by imaging studies (computed tomography and magnetic resonance imaging (MRI)) [30-33].

**Table 3:** RCC metastases to the nasal and paranasal regions

Sex/age at diagnosis	Location	Treatment of primary tumor	Interval between diagnosis of primary tumor and metastasis	Diagnosis of metastasis before primary treatment	Presenting symptoms	Metastases to other sites
f/65	Nasal	Nephrectomy	17 years	no	epistaxis	Thyroid, small intestine

m/58	Maxillary Sinus	Nephrectomy	11 years	no	lasting nasal obstruction	Lung

f/69	Nasal	Nephrectomy	NA	no	NA	Lung

f/60	Maxillary Sinus	Nephrectomy	10 years	no	Hyposmia, pain	-

f/87	Maxillary Sinus	Nephrectomy	eight years	no	epistaxis, nasal obstruction	-

m/71	Nasal	Nephrectomy	NA	no	epistaxis, nasal obstruction	Lung

N.A	Nose	-	Metastasis first	yes	NA	-

m/73	Ethmoid sinus	-	Metastasis first	yes	epistaxis	-

N.A	Ethmoid sinus	Nephrectomy	17 years	NA	epistaxis, nasal obstruction	-

f/45	Nasal Septum	-	NA	NA	NA	-

m/70	Nasosinusal	-	Metastasis first	yes	epistaxis	-

m/60	Maxillary Sinus	Nephrectomy	six months	NA	NA	-

f: female; m: male; NA: not available

### Tongue and tonsils

The tongue is a frequent target for RCC metastasis although isolated spread to the floor of the mouth is rare. Lesions in the tongue or floor of the mouth can cause severe pain, bleeding, difficulty eating and even complete oral obstruction. Unfortunately, oral cavity metastasis from RCC is usually a manifestation of widespread disease [34]. The literature review revealed 28 cases of RCC metastatic to the tongue. Out of these, only five cases presented initially with tongue metastases before the primary diagnosis of RCC [35,36].

As in most cases with distal metastases, prognosis for patients with lingual metastasis from RCC is poor. Treatment of tongue metastasis is usually palliative and aims to provide patient comfort by means of pain relief while preventing bleeding, infection and breathing difficulties. Surgical excision is recommended as palliative treatment with emphasis on preservation of tongue structure and function [34-42].

To the best of our knowledge only two cases of tonsil metastases from RCC have been reported during the last five years. Both patients were men, 61- and 76-years-old respectively, with known history of RCC and previously diagnosed bone and lung metastases [41,42].

### Thyroid gland

Although secondary involvement of the thyroid gland by RCC is uncommon, more than 150 cases of clinically recognized metastatic RCC to the thyroid have been reported in the English literature. Metastatic disease from the kidney to the thyroid gland can occur more than 20 years after nephrectomy, with an average time interval of approximately seven and a half years. Among them we found only five cases where metastasis to the thyroid gland was the first manifestation of RCC [43-45]. Some cases are depicted in Table 4[43-48].

**Table 4:** Cases of RCC metastases only to the thyroid gland

Sex/age at diagnosis	Treatment of primary tumor	Interval Between Diagnosis of primary tumor and metastases	Diagnosis of Metastasis before PT	Symptom
m/54	-	Metastasis first	yes	Dysphagia

m/73	Nephrectomy	eight years	NA	Painful mass, dyspnoea

f/61	Nephrectomy	seven years	no	N.A

m/72	Nephrectomy	17 years	no	swelling

f/66	Nephrectomy	16 years	no	N.A

m/61	Nephrectomy	eight years	no	swelling, cough

m/68	Nephrectomy	two years	no	asymptomatic

4 m/60-83, 6f/56-83	Nephrectomy in six cases		yes in four cases	

f: female; m: male; NA: not available

There are hypotheses explaining the relatively high incidence of metastases from the kidney to the thyroid gland. Although a popular theory claims that the proclivity of metastasis to the thyroid gland is related to its rich blood supply, some researchers have suggested that the abnormal thyroid gland is vulnerable to metastatic growth due to a decrease in oxygen and iodine content alteration [49].

Metastatic disease to the thyroid can manifest as breathing difficulties due to enlargement-swelling of the thyroid causing airway obstruction. Other symptoms include trouble or pain in swallowing, cough due to the vasogastric effect or a variety of symptoms of hypothyroidism [50]. Diagnosis is confirmed by thyroid scintigraphy, thyroid ultrasonography, and cytology of the material obtained through FNA [51].

### Heart

Isolated metastasis of RCC to the left ventricle of the heart is considered a rare incident. Historically, up to 10% of patients with RCC have tumor thrombus involving the renal vein and inferior vena cava and in up to 1% tumor thrombus extends into the right atrium. Metastasis to the left ventricle as to any other organ is possible via the hematogenous route. There have been rare reports of solitary late metastasis to the heart with the right ventricle being the preferred chamber involved. Metastasis to the heart may have two distinct origins and clinical features. The first is hematogenous, via the inferior vena cava, even in the absence of renal vein involvement; it is generally circumscribed and has a good prognosis after surgery. The second is through the intrathoracic lymphatic system, in the presence of disseminated disease, especially pulmonary metastasis. This type has a very poor prognosis [52].

There have been 10 reports within the last five years of cardiac metastases from RCC. In one case, a malignant pericardial effusion was the sole evidence of metastatic disease and was treated by radiation therapy [53]. In three cases [53-55] right atrial metastasis occurred, one of which displayed the absence of a vena cava tumor extension [54]. In three cases left ventricular metastasis occurred 18 to 23 years after nephrectomy [56-58]. In two cases there was right ventricular metastasis from RCC, which was diagnosed incidentally after an episode of syncope in the first case [59] and during the evaluation of hematuria in the second one [60]. Finally one case involved metastasis to the interventricular septum which caused cardiac paradox [61]. In Table 5 those cases are presented in detail.

**Table 5:** Cases with RCC metastasis to the heart, N

Sex/age at diagnosis	Location	Treatment of primary tumor	Interval between diagnosis of primary tumor and metastasis	Diagnosis of metastasis before primary treatment	Presenting symptoms	IVC involvement
f/67	Interverticular septum	Nephrectomy	three months	no	Dyspnea	N.A

m/50	Right atrium	Nephrectomy	six years	no	Budd-Chiari Syndrome	Yes

m/59	Right ventricle	-	Metastasis first	yes	Syncope	No

m/59	Pericardial, myocardial	Nephrectomy	NA	no	Cardiac arrest	No

m/69	Left ventricle	Nephrectomy	23 years	no	NA	NA

m/65	Left ventricle	Nephrectomy	20 years	no	NA	NA

m/50	Right atrium	Nephrectomy	one year	No	Raynaud-like phenomena, fatigue	No

f/59	Right atrium	-	zero	synchronous	NA	Yes

m/69	Left ventricle	Nephrectomy	18 years	No	Shortness of breath	No

m/55	Right ventricle	-	zero	synchronous	Hematuria	No

f: female; m: male; NA: not available

### Skin

Skin metastases of RCC are not easily identified because of the low suspicion index for these skin lesions, which usually mimic common dermatological disorders. Moreover in the majority of cases the pathogenesis of the skin lesion is not related to the primary tumor due to the long time interval since nephrectomy. Skin metastases have been reported to occur in around 3% of renal tumors. They are more common in males. Several cases of calvarial metastases as secondary lesions from RCC have been reported in the literature, but only five cases have been described concerning calvarial mass as the first clinical presentation of metastatic RCC [62-64].

Skin metastases mainly occur in the head, neck and trunk, in that order. Skin metastases from RCC occur in most patients at a late stage of the disease, usually years after nephrectomy for an organ-confined tumor. However in some cases they may occur even before the renal tumor is diagnosed [65]. Skin metastases are usually considered late manifestations of the disease, bearing a poor prognosis that is associated to synchronous visceral metastases in up to 90% of cases, resulting in tumor-specific survival of usually shorter than six months [66-73].

### Ovaries-Uterus-Testis

Metastasis to the ovaries is thought to occur by retrograde venous embolization through the renal vein to the ovarian vessels. In an autopsy study, ovarian metastasis was found in 0.5% of cases of renal cancer. Metastasis through this pathway exploits the unique anatomy of the left renal and ovarian veins. It mandates incompetent gonadal veins to allow for retrograde venous blood flow. As a matter of fact, two thirds of reported cases arose from a left-sided lesion. Thus, it appears that the hallmark for the renal-ovarian axis is its unique venous anatomy. Only 21 cases of metastasis to the ovary from RCC have been reported in the literature (eight cases in the last five years). Out of these, 17 cases were metastasis of RCC of clear cell type. Six of these cases were diagnosed as primary ovarian clear cell cancer, while renal primary was diagnosed only after extensive investigations [74-81].

Regarding the vagina and uterus, to our knowledge, there is one report in the literature of metastatic oncocytic papillary RCC to the endometrium in an 89-year-old woman presented with vaginal bleeding, one of vaginal metastasis from RCC and another one of cervical carcinoma in a 45-year-old woman [80,81].

With regard to metastatic testicular involvement, the incidence of secondary testicular tumors ranges from 0.3% to 3.6% with the most frequent primary site being the prostate [82]. Intrascrotal metastasis arising from RCC has also been reported [83]. The pathologic diagnosis of RCC metastatic to the testis almost always reveals a clear cell tumor pathology [84]. To our knowledge only six cases of testicular metastases from RCC have been reported within the last five years, in one of the six, there was a contralateral chromophobe RCC metastatic to the testis, six years after nephrectomy [85,86].

### Muscle and joints

RCC metastatic to muscles is a very rare incident indeed. According to Satake *et al*., up until 2009 only 32 cases of skeletal muscle metastasis from RCC had been reported; our search added another three. The fact is that there are very few cases with muscle metastasis from RCC with each metastatic location comprising a unique case report [87,88].

Only three cases of acute monarthritis secondary to asymptomatic RCC have been described. The patients were initially diagnosed with septic arthritis. However the finding of hot spots on isotope bone scans and biopsy samples showing secondary neoplasms confirmed the lesions to represent metastatic sites of RCCs. MRI has been proven helpful in delineating the features and extent of the muscle invasion by the tumor [88,89].

## Conclusions

RCC represents a potentially lethal cancer that is associated with aggressive behavior and has a propensity for metastatic spread. The patterns of metastases from RCCs are not yet defined with accuracy and, as a result, RCC has been associated with rare metastatic sites and occasionally atypical presenting symptoms from disseminated disease and distant metastatic sites.

The present review has focused on these rare incidences of metastatic spread of RCC to uncommon sites and organs both at the time of diagnosis of the primary tumor but also years after radical nephrectomy. This review is mainly based on published case reports relevant to the metastatic potential of RCC. This fact further highlights the significance of case reporting, especially in oncology where clinical trials or even large case series are not always available, as Dib *et al*. have very elegantly pointed out [90]. The contribution of case reporting should not be underestimated since many of our classical clinical teachings have originated from the observation of isolated "case reports".

## Competing interests

The authors declare that they have no competing interests.

## Authors' contributions

PS and LC were responsible for the concept of the article and reviewed the final draft, LM reviewed the relevant papers and wrote the first draft of the paper. All authors have read and approved the final manuscript.
